# Genome-Wide Analysis of Müller Glial Differentiation Reveals a Requirement for Notch Signaling in Postmitotic Cells to Maintain the Glial Fate

**DOI:** 10.1371/journal.pone.0022817

**Published:** 2011-08-02

**Authors:** Branden R. Nelson, Yumi Ueki, Sara Reardon, Mike O. Karl, Sean Georgi, Byron H. Hartman, Deepak A. Lamba, Thomas A. Reh

**Affiliations:** Department of Biological Structure, University of Washington, Seattle, Washington, United States of America; Universidade Federal do Rio de Janeiro, Brazil

## Abstract

Previous studies have shown that Müller glia are closely related to retinal progenitors; these two cell types express many of the same genes and after damage to the retina, Müller glia can serve as a source for new neurons, particularly in non-mammalian vertebrates. We investigated the period of postnatal retinal development when progenitors are differentiating into Müller glia to better understand this transition. FACS purified retinal progenitors and Müller glia from various ages of Hes5-GFP mice were analyzed by Affymetrix cDNA microarrays. We found that genes known to be enriched/expressed by Müller glia steadily increase over the first three postnatal weeks, while genes associated with the mitotic cell cycle are rapidly downregulated from P0 to P7. Interestingly, progenitor genes not directly associated with the mitotic cell cycle, like the proneural genes *Ascl1* and *Neurog2*, decline more slowly over the first 10–14 days of postnatal development, and there is a peak in Notch signaling several days after the presumptive Müller glia have been generated. To confirm that Notch signaling continues in the postmitotic Müller glia, we performed *in situ* hybridization, immunolocalization for the active form of Notch, and immunofluorescence for BrdU. Using genetic and pharmacological approaches, we found that sustained Notch signaling in the postmitotic Müller glia is necessary for their maturation and the stabilization of the glial identity for almost a week after the cells have exited the mitotic cell cycle.

## Introduction

Glial cells are generated at the end of histogenesis in the developing CNS [1]. In many regions of the CNS, the progenitors become glia after the process of histogenesis is complete. For example, in the cerebral cortex, many radial glia transform into astrocytes at the end of histogenesis (for review see [2]). In the retina, a similar process occurs, and the Müller glial cells are among the last cell types generated during histogenesis (for review see [3]).

Several signaling systems have been implicated in this transition from multipotent progenitors to Müller glia. The Notch signaling pathway, along with downstream effectors, Hes1, Hes5 and Hesr, plays a key role in Müller glial differentiation [4], [5], [6], [7], as it does in astrocyte differentiation elsewhere in the CNS [8]. Over-expression and knockout studies support the model that prolonged activation of the Notch pathway leads to acquisition of the Müller glial fate by the retinal progenitors [9], [10], [11]. Egfr stimulation is also important in the generation of Müller glia from progenitors and in the regulation of their proliferation; over-expression of constitutively active Egfr can drive progenitors to the Müller glial fate [12], as can prolonged treatment with EGF [13]. Loss of Egfr during development leads to a reduction in proliferation of the progenitor cells [14]. CNTF and LIF also play a key role in stimulating the differentiation of Müller glia [15], [16], as they do for astrocytes in the rest of the CNS [17]. Despite the wealth of information concerning these signaling molecules and the acquisition of the Müller fate, it is not clear how they are integrated during this transition. This is important because in non-mammalian vertebrates, retinal damage causes these cells to regain their neurogenic potential and serve as a substrate for regeneration [18].

To gain insight into the mechanisms that control this key step in retinal development, we carried out a series of cDNA microarray studies on the progenitors and developing Müller glia. We purified the progenitor and Müller glial cells using a Hes5-GFP line of mice that has been previously used to purify neural progenitor cells [19]. After FACS purification, RNA extraction and Affymetrix array analysis, we used unsupervised K-medians clustering, to generate several informative groups. Over the first three postnatal weeks, we found a steady increase in genes previously shown to be enriched in Müller glia, confirming and extending previous studies of the Müller glial transcriptome [19], [20]. In addition, we found a rapid decline in cell cycle related genes between P0, when progenitors are actively proliferating, and P7, when genesis of retinal cells is complete in the mouse. By contrast, factors known to be important for glial differentiation continue to be expressed through the second postnatal week. This is particularly evident in genes associated with Notch signaling, and confirmation with combined *in situ* hybridization and BrdU shows that Notch signaling remains high in the Müller cells for 4–5 days after they have exited the mitotic cycle, suggesting that sustained Notch signaling in the Müller glia is necessary for their maturation and the stabilization of the glial identity. We tested this possibility by inhibiting Notch signaling in the Müller glia between postnatal day 7 and day 12 and found that this caused an increase in proneural gene expression in these cells. In addition, genetic activation of Notch in retinal progenitors promotes glial gene expression only after the cells have exited the cell cycle. We conclude that neurogenic potential persists for nearly a week after the Müller glia have exited the cell cycle, and that Notch signaling is required for the maintenance of the Müller glial fate during this period when the other gliogenic signals are upregulated.

## Results

### Hes5-GFP is expressed in progenitors and Müller glia

In order to purify the progenitors and Müller glia from the developing retina, we took advantage of the fact that these two cell populations express Hes5 [6]. The construct used to generate the Hes5-GFP mice contains a 3 kb portion of the *Hes5* gene, including 1.6 kb of the 5-prime flanking region, with eGFP cloned into the endogenous translational start site [21]. Previous characterization of this line demonstrated that GFP is reliably expressed in regions known to express Hes5 in the developing brain [19] and the inner ear sensory epithelia. In addition, Hes5-GFP in these mice is inducible by Notch1 activation and absent from Notch1-deficient embryos [21].

**Figure 1:**
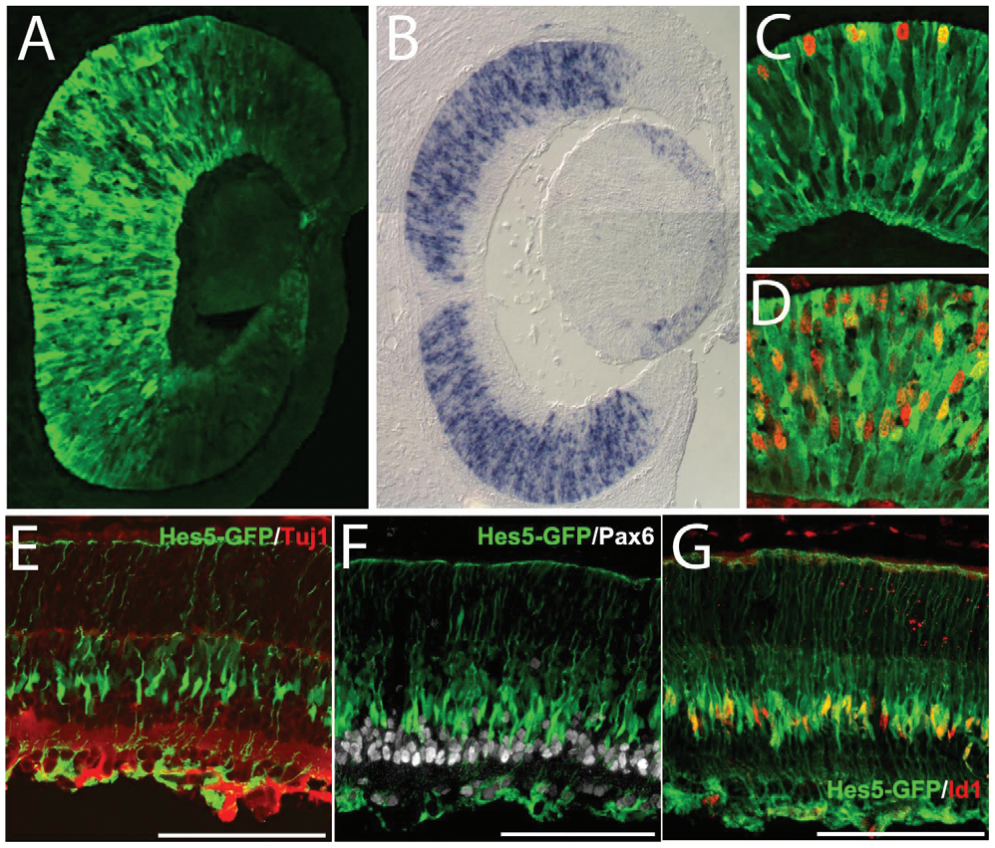
Hes5-GFP expression in developing retina. (A) Hes5-GFP in the E12.5 mouse retina. (B) in situ hybridization for *Hes5* mRNA at E13.5. (C,D) Hes5-GFP expressing cells in the E12.5 day retina co-express progenitor markers, PH3 (C) and BrdU (D). (E–G) Hes5-GFP expression at postnatal day 7 is confined to the inner nuclear layer in the maturing Müller glia (Id1+; G)), and is not expressed in the Pax6+ or TuJ1+ inner retinal neurons (E and F, respectively).

**Figure 2:**
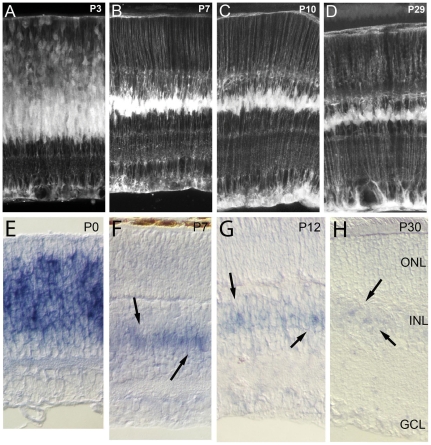
The expression of Hes5-GFP is similar to that of *Hes5* mRNA at all ages. (A,E) Hes5 mRNA and Hes5-GFP are expressed throughout the progenitor zone. (B,F) At postnatal day 7, postmitotic Müller glia express Hes5-GFP and *Hes5* mRNA. (C,G) At postnatal day 12, the expression of *Hes5* mRNA is markedly reduced, as is the expression of Hes5-GFP. (D,H) In mature retina, the expression of *Hes5* mRNA remains in the Müller glia, but the levels are so low that only faint labeling can be observed with the in situ, while the Hes5-GFP is still clearly visible throughout the Muller cells.

As described by Basak and Taylor [21], in these mice, Hes5-GFP expression is restricted to the progenitors in embryonic retina. Figure 1A shows expression of the Hes5-GFP in the E12.5 mouse retina compared with a similar age in situ hybridization for *Hes5* mRNA (Figure 1B). The expression is confined to the progenitors cells in the neuroblastic layer in the central retina. The Hes5-GFP expressing cells in the E12.5 day retina co-express progenitor markers, PH3 (Figure 1C) and BrdU (Figure 1D). The GFP continues to be expressed in the progenitor cells in the neonatal retina (see below) and corresponds closely with the Hes5 mRNA (Figure 2E). By postnatal day 7, however, the expression of Hes5-GFP is considerably reduced and is now confined to the inner nuclear layer (Figure 1(E–G), in the maturing Müller glia (Id1+; Figure 1G)), and not in the Pax6+ or TuJ1+ inner retinal neurons (Figures 1 E and F, respectively) or outer nuclear layer. Nevertheless, in the early postnatal ages (P0–P3), there is a low level persistence of the GFP in the photoreceptors and bipolar cells generated by the postnatal progenitors, due to the stability of the GFP.

The expression of Hes5-GFP is similar to that of *Hes5* mRNA at all ages we examined (Figure 2). As noted above, for the first three postnatal days, when the multipotent progenitors are still in the mitotic cell cycle in the central retina, Hes5 mRNA and Hes5-GFP are expressed throughout the progenitor zone (Figure 2A and E). By postnatal day 7, retinal histogenesis is complete in the central retina, though the postmitotic Müller glia continue to express Hes5-GFP and *Hes5* mRNA (Figure 2B and F). By postnatal day 12, the expression of *Hes5* mRNA is markedly reduced, as is the expression of Hes5-GFP (though the gain has been increased to show the details of the Müller glia in Figure 2C and D). In mature retina, the expression of *Hes5* mRNA remains in the Müller glia, but the levels are so low that only faint labeling can be observed (Figure 2H). Nevertheless, the expression of Hes5-GFP is still clearly visible in the Müller glia, even at P29, though as noted the gain of the microscope was increased over that required for acquisition of the images at P3 and P7. It is also important to note that we did not find expression of the Hes5-GFP in the retinal astrocytes, consistent with Basak and Taylor [21] who failed to find Hes5-GFP expression in astrocytes in other regions of the central nervous system. By postnatal day 7, retinal astrocytes have migrated across the vitreal surface of the retina in the mouse [22], and although it is difficult to completely rule out Hes5-GFP expression in the astrocytes, we did not detect Hes5 mRNA expression in cells at the vitreal surface near the GCL (Figure 2).

**Figure 3:**
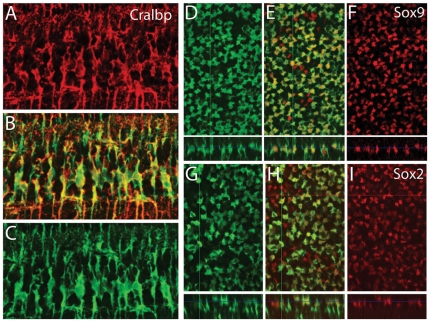
Double-label immunofluorescence staining with known Müller glial markers, Cralbp, Sox9 and Sox2 with Hes5-GFP in adult mouse retinas. (A–C) Retinal section showing correspondence between Cralbp (red) and Hes5-GFP (C,). (D–I) Confocal optical sections in the xy and xz planes showing double-labeling with Muller glial nuclear markers Sox9 (D–F, red) and Sox2 (G–I, red). Note that not all Sox9+ or Sox2+ cells express the Hes5-GFP; the reporter is expressed in most, but not all, Muller glia.

To further validate the Müller glial specific expression of Hes5-GFP, we also carried out double-label immunofluorescence staining with known Müller glial markers, Cralbp, Sox9 and Sox2 in adult retinas (Figure 3). There was near complete overlap between the Cralbp and Hes5-GFP (Figure 3A–C); however, while all Hes5-GFP+ cells were Sox2+ and Sox9+, we found that the Hes5-GFP did not label all the Müller glia; there were occasional Sox2+ and Sox9+ cells that were not Hes5-GFP+. The incomplete, somewhat mosaic expression of the Hes5-GFP was also true of progenitors in the embryonic and newborn retinas, though in most animals, fewer than 10% of the progenitors in the retina were not labeled with the reporter.

### Microarray analysis of FACS purified retinal progenitors and Müller glia

**Figure 4:**
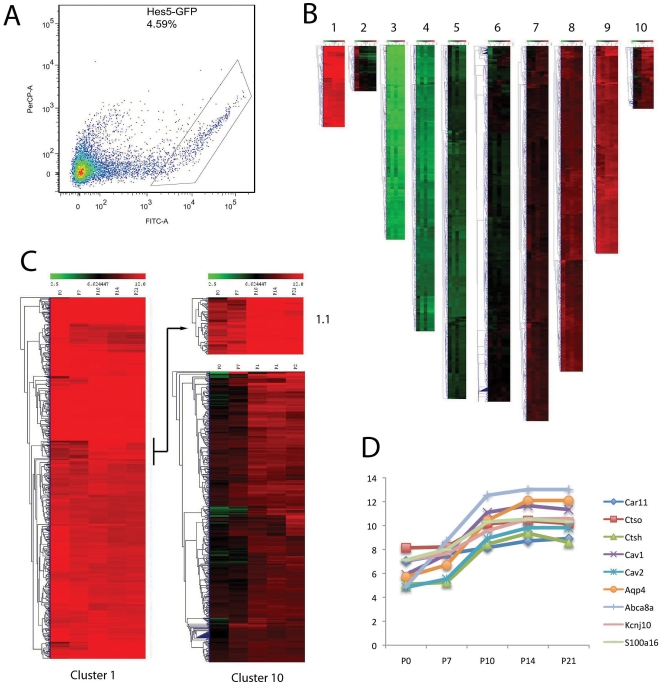
FACS purified Muller glia subjected to Affymetrix analysis. (A) Hes5-GFP expressing cells were separable from non-expressing cells, with the box outlining the gate for selection of the positive cells. (B) The gene expression levels (log transformed) across the five ages of retinal development are shown as heat maps. (C,) Clusters 1 (with 3% of the total genes) and 10 (with 3% of the total) contained previously characterized Müller glial genes. (D) Graph of the expression levels of 9 genes previously reported to be enriched in mature Müller glia,; all of these genes show a clear increase between P7 and P10 as the Müller glia mature.

To selectively analyze the gene expression of progenitors and Müller glia over the period when the Müller cells are maturing, we took advantage of the fact that Hes5-GFP was expressed in these two populations. The retinas from Hes5-GFP mice at postnatal ages P0, P7, P10, P14, P21 were dissociated and the GFP+ cells were isolated by FACS. The Hes5-GFP expressing cells had a high enough level of expression at all the ages we examined so that we could sort them from the non-GFP expressing cells (Figure 4A). The number of GFP+ cells from mature retinas were close to those expected for the number of Müller glia in the mouse retina (approximately 5%; [23]); the number of GFP+ cells isolated from the P0 retinas was higher, approximately 40%, again similar to estimates of the percentages of progenitors in the newborn mouse retina. The FACS purified cells from P21 retinas were assessed for purity by labeling with a Müller glial marker, vimentin, and >95% were positive. The RNA was isolated and subjected to microarray and cluster analysis as described in the Methods.

Unsupervised K-medians clustering of all genes on the array (28,853) was used to generate 10 clusters, and the gene expression levels (log transformed) across the five ages of retinal development are shown as heat maps in Figure 4 and as Table S1 and Figure S1. Clusters 1,2,7–10 contained genes that had expression levels for at least one age that were above the threshold; these genes constituted 44% of the genes on the array. The genes in clusters 3–6 did not reach the threshold level of expression for any age, and so these clusters were not analyzed further. In general, Clusters 1 (with 3% of the total genes) and 10 (with 3% of the total) contained genes that are either highly expressed at all ages (Cluster 1), or tend to increase over the first three postnatal weeks (Cluster 10) in the maturing Müller glia. Clusters 2, 7 and 8 contained genes that trend down over the period of analysis, and many of the genes in Cluster 2, for example, are related to the mitotic cell cycle (see below). Clusters 7 and 8 contain 15% and 13% of the total genes, respectively, and decline over the period of analysis. Cluster 9, with 8% of the total genes, contains genes with levels of expression close to that of Cluster 1, though the changes in expression over the first three postnatal weeks is more complex in Cluster 9, with expression levels in some subclusters increasing with age and others declining.

When the individual clusters were further analyzed for previously characterized genes that are enriched in progenitors and/or Müller glia, some patterns emerged. Cluster 1 contained the most highly expressed genes across all five ages; these are genes that are highly expressed in progenitors and also in Müller glia. Most genes that are expressed at lower levels in progenitors, but steadily increase with Müller glial maturation were assigned to Cluster 10. A further analysis of Cluster 1 also revealed a sub-cluster (1.1) that showed a similar pattern of gene expression as those in Cluster 10, low in progenitors and increasing with glial maturation (Figure 4C). Many previously characterized Müller glial markers were present in Clusters 1 and 10. Cluster 1, for example, contains, *Vimentin*, *Sox9*, *Sox2*, Glutamine synthetase (*Glul*), and carbonic anhydrase (*Car2*). Cluster 10 also contains previously characterized Müller glial expressed genes, including *CyclinD3*, *Egfr*, *S100a1*, *S100a16*, *Bmpr1b*, and *Cntfr*. Table 1 shows the genes that are expressed in either Cluster 1 or 10, which previously were reported in Müller glia. Most of the genes identified from single cell microarray analysis by Roesch et al, [19], as well as those identified in a proteomic analysis of purified Müller glia [20] were expressed in Clusters 1 and 10. Figure 4 D shows a graph of 9 genes previously reported to be enriched in mature Müller glia, which were present in Cluster 10; all of these genes show a clear increase between P7 and P10 as the Müller glia mature. We also found that many genes that were previously shown by others to be selectively expressed in astrocytes were also present in Clusters 1 and 10 (Table 1), reflecting the similarity between astrocytes and Müller glia. The expression of these known astrocytic markers in our samples is not likely to be due to contaminating astrocytes in our FACS purified samples, since as noted above, Hes5-GFP is not expressed in retinal astrocytes; moreover, we did not detect GFAP expression in our Hes5-GFP FACS sorted cells, even though this is one of the most highly expressed genes in astrocyte gene arrays, and a marker for retinal astrocytes [22]. We also examined the data for evidence of contamination from other retinal cell populations, particularly rod photoreceptors, amacrine cells and bipolar cells, which together constitute over 90% of the retinal cells. By comparison with other recent microarray studies of these cell types, we found that fewer that Cluster 10 contained several of the most highly expressed rod photoreceptor genes [24], but altogether these constitute only 2% contamination from rod photoreceptor genes, and less than 0.2% contamination from amacrine and bipolar genes. Cluster 1 had only a single gene of the top 40 highly expressed in rod photoreceptors (less than 0.2% contamination), and none of the genes identified as bipolar or amacrine cell specific [24], [25], [26]. These results validate our purification and analysis approach, and suggest that the other genes in Clusters 1 and10 might also be enriched in Müller glia.

**Table 1:** Genes in Clusters 1 and 10 from the present analysis of Hes5-GFP+ cells from postnatal and adult mice that were previously identified as enriched in Müller glia and/or in astrocytes validates our purification method and highlights the similarity between Müller glia and astrocytes.

Müller Genes		Astrocyte Genes	
*Abca8a*	Roesch et al, 2008	*Acot1l*	Cahoy et al, 2008
*Aldh1a1*	*Hauck et al*, *2003*	*Aldh1l1*	Cahoy et al, 2008
*Aldoc*	*Hauck et al*, *2003*	*Aldoc*	Cahoy et al, 2008
*Anax6*	*Hauck et al*, *2003*	*Anax1*	Obayashi et al, 2009
*ApoE*	Amaratunga et al, 1996	*Aqp4*	Cahoy et al, 2008
*Aqp4*	Ottersen et al, 1998	*Atp1a2*	Cahoy et al, 2008
*Car2*	Roesch et al, 2008	*Bmpr1b*	Cahoy et al, 2008
*Cav*	Roesch et al, 2008	*Cfi1*	Obayashi et al, 2009
*Ccnd3*	Dyer and Cepko, 2000	*Chrdl1*	Cahoy et al, 2008
*Chx-10*	Rowan et al, 2004	*Ctgf*	Obayashi et al, 2009
*Ckb*	*Hauck et al*, *2003*	*Ctsh*	Obayashi et al, 2009
*CRLBP-1*	Bunt-Milam and Saari, 1983	*Dio2*	Cahoy et al, 2008
*Ctsh*	Roesch et al, 2008	*F3*	Cahoy et al, 2008
*Dbi*	Roesch et al, 2008	*Gadd45b*	Obayashi et al, 2009
*Dkk3*	Blackshaw et al, 2004	*Id2*	Obayashi et al, 2009
*Egfr*	Close et al, 2006	*Mertk*	Cahoy et al, 2008
*Eno1*	*Hauck et al*, *2003*	*Pcsk5*	Obayashi et al, 2009
*Gabra1*	Biedermann et al, 2002	*Pla2g7*	Cahoy et al, 2008
*Glul*	Riepe and Norenburg, 1977	*Ppp1r3c*	Cahoy et al, 2008
*Gnai2*	Roesch et al, 2008	*Prodh*	Cahoy et al, 2008
*Gnb1l*	Roesch et al, 2008	*Slc14a1*	Cahoy et al, 2008
*GPR37*	Roesch et al, 2008	*Slc1a3*	Cahoy et al, 2008
*Gstt1*	*Hauck et al*, *2003*	*Sulf1*	Obayashi et al, 2009
*Itm2b*	Roesch et al, 2008		
*Kcnj10*	Newman and Reichenbach		
*Pebp1*	*Hauck et al*, *2003*		
*Pgk1*	*Hauck et al*, *2003*		
*Prdx2*	*Hauck et al*, *2003*		
*S100a16*	Terenghi et al, 1983		
*Sod1*	*Hauck et al*, *2003*		
*Sox2*	Blackshaw et al, 2004		
*Sox9*	Poche et al, 2008		
*Synpr*	Roesch et al, 2008		
*Vimentin*	Pixley and de Vellis, 1984		

**Figure 5:**
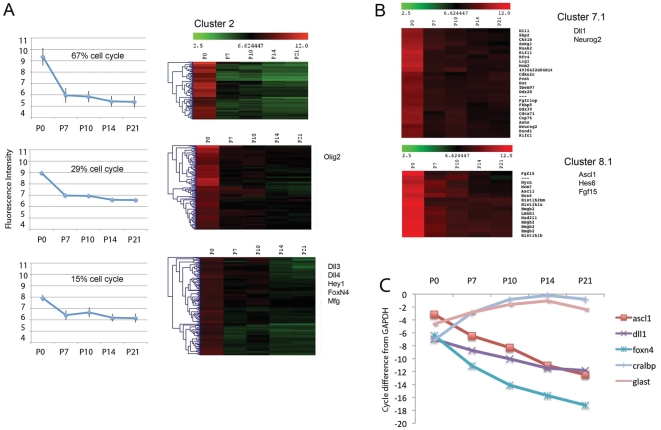
Heat maps of hierarchically sorted genes as a function of age, showing genes that are expressed highly in progenitors and at much lower levels in the Müller glia. (A) Cluster 2 contains genes with Gene Ontology terms associated with the mitotic cell cycle; some retinal progenitor genes are also associated with this cluster, particularly subCluster 2.3. (B) The expression levels of genes of two additional subclusters, Cluster 7.1 and Cluster 8.1 are shown as heat maps. Cluster 7.1 contains the progenitor genes, *Neurog2* and *Dll1*, while Cluster 8.1 contains progenitor genes *Ascl1*, *Hes6*, *n-Myc* and *Fgf15*. (C,) RT-PCR confirmation of expression profiles for some of the progenitor genes from FACS purified cells across the five ages.

Another informative cluster was Cluster 2 (Figure 5A). This cluster contains most genes that are expressed highly in progenitors and at much lower levels in the Müller glia. Cluster 2 can be further subdivided into three subclusters that show differences in expression from P0 to P7. Cluster 2.1, for example, contains genes that change most dramatically between P0 and P7, and the majority of the genes in this subcluster have Gene Ontology terms associated with the mitotic cell cycle. Genes in Subcluster 2.2 show less dramatic changes in expression between P0 and P7, but again, many of these genes are also associated with cell cycle and its regulation; however, the progenitor gene, *Olig2*, has an expression profile that put it in this group. Subcluster 2.3 contains genes that are expressed in progenitors and are not so rapidly down-regulated at P7. Many progenitor genes, including *Dll3*, *Dll4*, *Hey1*, *FoxN4*, and *Mnfg* are contained in this subcluster (Table 2). Of the more than 400 genes in Cluster 2, nearly 140 are known to be associated with the mitotic cell cycle in other cells (Table S2) and most of these have not been previously shown to be expressed in retinal progenitors [27], and so this analysis greatly expands the list of candidate regulators of retinal progenitor proliferation.

**Table 2:** Genes previously shown to be important for neurogenesis are expressed in Clusters 2,7,8,and 9.

Progenitor Genes			
Cluster 2	Cluster 7	Cluster 8	Cluster 9
*Dll3*	*Dll1*	*Ascl1*	*Id1*
*Dll4*	*Neurog2*	*Fabp7*	*Id3*
*Foxn4*	*Numbl*	*Fgf15*	*Id4*
*Heyl*	*Numb*	*Hes6*	*Lfng*
*Mfng*	*Notch3*	*Hey1*	*Lhx2*
*Olig2*	*Six3*	*Maml1*	*Rax*
*Sfrp2*	*Zic1*	*Nes*	
	*Zic2*	*Rbpj*	

The expression of all of these genes is downregulated in the first two postnatal weeks, but the genes in Cluster 2 decline rapidly between P0 and P7, while those in the other clusters decline more slowly.

**Figure 6:**
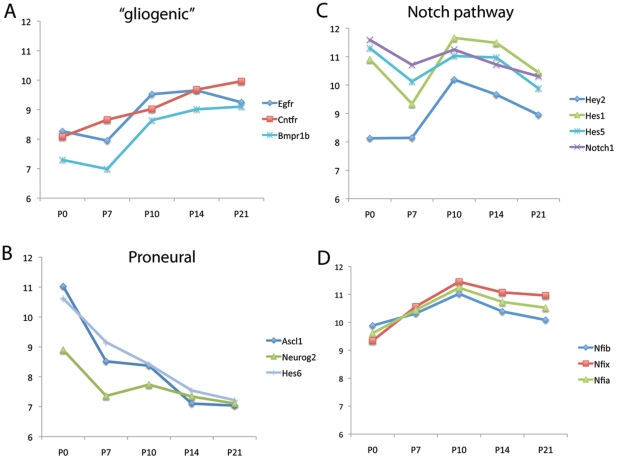
Line graphs of the expression levels of specific genes as a function of age in the Hes5-GFP FACS purified cells. (A) The receptors for the three main “gliogenic” receptors, *Cntfr*, *Egfr* and *Bmpr1b*, steadily increase in expression from postnatal day 0 to postnatal day 21. (B) The proneural transcription factors, *Ascl1*, *Hes6*, and *Neurog2*, all steadily decline from postnatal day 0 to P21. (C,) The Notch signaling components show more complex patterns of expression; Notch1 declines from P0 to P21, but the downstream effectors, *Hey2/Hesr2*, *Hes5* and *Hes1* reach their peak on postnatal day 10. (D) The nuclear factors I-a, -b and -x (*Nfia/b/c/x*) reach their peak on postnatal day 10.

Many of the known progenitor genes were not contained in Cluster 2, but were present in Clusters 7 and 8. Genes in these clusters generally trended down over the period we examined, showing high expression in progenitors and declining in many cases to very low levels by postnatal day 14 or 21. Genes associated with neurogenesis that were present in these clusters are shown in Table 2. Many of the progenitor-specific genes, like *Neurog2*, *Zic1/2*, *Ascl1*, *Hes6*, *Nestin*, and *Fgf15* are present in these clusters. The expression of two subclusters, Cluster 7.1 and Cluster 8.1 are also shown in Figure 5B. Cluster 7.1 contains the progenitor genes, *Neurog2* and *Dll1*, while Cluster 8.1 contains progenitor genes *Ascl1*, *Hes6*, *n-Myc* and *Fgf15*. RT-PCR confirmation of these expression profiles for some of the progenitor genes across the five ages is shown in Figure 5C, and a summary of the expression profiles of the proneural genes from the array data is shown in Figure 6B. In general, as expected, these genes all trend down over the period we analyzed, expressed most highly in progenitors, less so at P7 and to adult levels by postnatal day 14. However, the somewhat gradual decline of the proneural genes, when compared with the genes involved in mitotic cell cycle (Cluster 2) was somewhat surprising, and we validate this below.

As noted in the Introduction, several different signaling systems have been shown to promote gliogenesis and differentiation in the developing nervous system, including BMP, EGF, CNTF/LIF and Notch, and it was interesting to determine the level of expression in the receptors for these signals during the transitional period between progenitors and Müller glia. The data from each age for the three main “gliogenic” receptors, *Cntfr*, *Egfr* and *Bmpr1b*, are shown in Figure 6A. The receptors for each of these signals steadily increase in expression from postnatal day 0 to postnatal day 21 (Figure 6 A). By contrast, the proneural transcription factors, *Ascl1*, *Hes6*, and *Neurog2*, all steadily decline from postnatal day 0 to P21 (Figure 6B). The Notch signaling components show more complex patterns of expression. Notch1 declines from P0 to P21 (Figure 6C), but the downstream effectors, *Hey2/Hesr2*, *Hes5* and *Hes1* reach their peak on postnatal day 10. Negative regulators of basic helix-loop-helix (bHLH) transcription factors, the Id proteins, have also been shown to be gliogenic when over-expressed in CNS progenitors, possibly through inhibiting the autoinhibition of Hes1 on its own promoter [28], or alternatively by antagonizing the proneural bHLH transcription factors. All four of the members of the Id family are expressed in the developing retina, and reach their peak during the period from P10 to P14 (Table S4). Another group of “pro-glial” transcription factors, the nuclear factors I-a, -b and -x (*Nfia/b/c/x*), are also expressed in the developing retina, and also reach their peak on postnatal day 10 (Figure 6D). Thus, it appears from the array data that a coordinated gliogenic period persists, and even reaches a maximum several days after the cells have exited the mitotic cell cycle.

### The Notch pathway remains active in postmitotic Müller glia and stabilizes the glial fate

**Figure 7:**
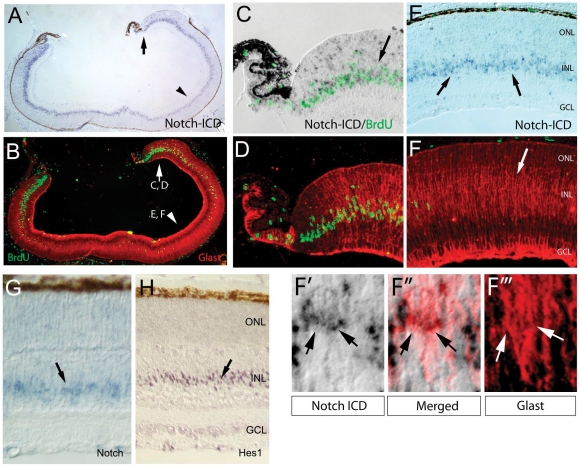
A combination of BrdU labeling and immunohistochemistry for active Notch signaling shows Notch remains active after the Muller glia have become postmitotic and begun their differentiation. Immunolebeling for active Notch (Notch-ICD; A,C,E, F, F′, F″″, F″″″). An injection of BrdU at postnatal day 5, followed by sacrifice 2 hours later, results in BrdU incorporation in S-phase cells in the peripheral fourth to one third of the retina (Green, C–F). (C,F) The Müller glia in the central retina have already begun their differentiation, as indicated by the strong labeling for Glast, while the progenitor cells in the peripheral retina have only a low level of Glast (C,D). The Notch pathway is active both in the peripheral retinal progenitors (BrdU+;A, C), but also in the central Müller glia (BrdU−/Glast+; A, E, and F-F′″). (G) In situ hybridization at P7 for *Notch* mRNA expression (H) Hes1 immunohistochemistry localizes to the Muller glia in the INL. All panels except G and H are from the same section, with the arrows and arrowheads in panels A and B pointing out the regions at higher magnification in panels C/D and E/F, respectively. Panels F-F″″″ show the region identified by the arrow in panel F, whereas the arrows in F-F″″″ point to Muller cells (Glast+) that also express Notch-ICD.

The results of the array analysis demonstrated that several downstream effectors of the Notch pathway are expressed in FACS purified Müller glia after these cells have become postmitotic. We confirmed this using a combination of BrdU labeling (to distinguish the progenitors and postmitotic cells) and immunohistochemistry for the active form of the Notch receptor (the Notch-ICD; Figure 7). An injection of BrdU at postnatal day 5, followed by sacrifice 2 hours later, results in BrdU incorporation in S-phase cells in the peripheral fourth to one third of the retina; at this stage in development, the progenitors have all terminally exited the cell cycle in the central retina (Figure 7). The Müller glia in the central retina have already begun their differentiation, as indicated by the strong labeling for Glast (Figure 7F), while the progenitor cells in the peripheral retina have only a low level of Glast (Figure 7D). Thus, the BrdU and Glast labeling correlate well with the transition between the progenitor and Müller glial state. When the same sections are examined for expression of Notch-ICD, the Notch pathway is active both in the peripheral retinal progenitors (BrdU+;Figure 7 A, C), but also in the central Müller glia (BrdU−/Glast+; Figure 7 A, E, and F-F′″). The in situ hybridization at P7 also shows clear *Notch* mRNA expression (Figure 7G) and scattered cells in the INL express the Notch ligand, *Dll1* (not shown). Hes1 immunohistochemistry (Figure 7H) and *Hes5* in situ hybridization (Figure 2F), also label a band of cells across the INL, similar to the Notch expression. Together, these data demonstrate the presence of active Notch signaling in the postmitotic Müller glia.

**Figure 8:**
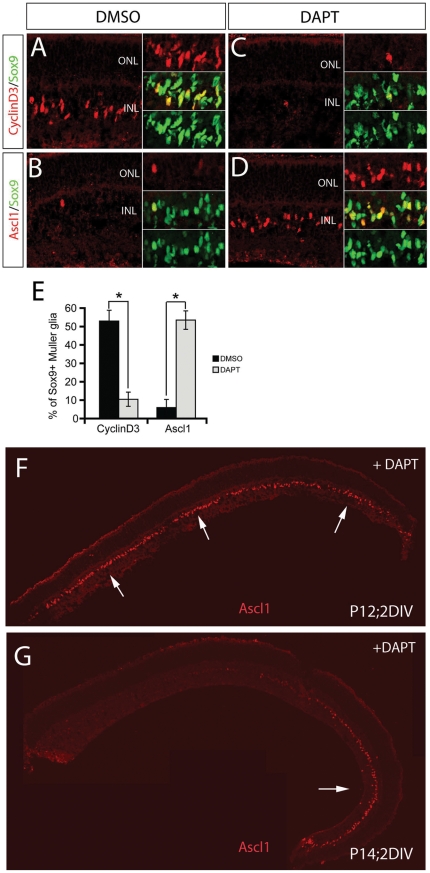
Inhibition of Notch signaling prevents glial maturation. Retinas from postnatal day 12 mice treated with DAPT for two days, and then sectioned the retinas and labeled for Ascl1 (A,C, red), and the Müller glial marker, CyclinD3 (B,D,red) and Sox9 (green). When Notch signaling was blocked with DAPT, there was a striking reduction in the number of Ccnd3+/Sox9+ cells, when compared with the DMSO-treated control retinas, but an increase in Ascl1+/Sox9+ cells. (E) This effect is quantified for the P12 retina. (F,G) Comparison of the effect of DAPT on Ascl1 expression (red) at P12 and P14 shows that by postnatal day 14, the Müller glial fate was not affected by DAPT, except at the far peripheral retina,(arrow).

We hypothesized that maintained Notch signaling in the postmitotic Müller glia was necessary for their differentiation and tested this hypothesis by blocking Notch with a small molecule gamma-secretase inhibitor, DAPT [29], [30]. We treated retinas from postnatal day 8 and postnatal day 12 mice with DAPT for two days, and then sectioned the retinas and labeled them with antibodies against the progenitor marker, Ascl1, and the Müller glial marker, CyclinD3 (*Ccnd3*) (Figure 8A–D). The sections were co-labeled with Sox9, and since this is expressed in both progenitors and Müller glia, we could control for any effects on the overall number of progenitors/glia. The use of nuclear markers for the cells allowed us to better quantify the effects of the DAPT. When Notch signaling was blocked with DAPT, there was a striking reduction in the number of Sox9+ cells that expressed the Müller glial marker, Ccnd3, when compared with the DMSO-treated control retinas (Figure 8A,C,and E). At the same time, the DAPT caused the induction of Ascl1 in the Sox9+ Müller glia (Figure 8B,D,and E). This effect was observed at both postnatal day 8 and postnatal day 12 (Figure 8F), but no longer occurred in most of the Müller glia at postnatal day 14, by which time the Müller glial fate was not affected by DAPT (except at the far peripheral retina, Figure 8G). Another gene specific for neural progenitor cells in the retina is *Neurog2* [31]. We used the Neurog2-tamoxifen-inducible creER knock-in mice (Zirlinger et al, 2002 crossed to the ROSA-lacZ or ROSA-YFP reporter line (Neurog2^ki-creER/YFP^) to confirm that DAPT treatment of P12 Müller glia promotes the neurogenic state; when retinas from these mice were cultured in the presence of tamoxifen and treated with DAPT, as late as postnatal day 12, we found that the *Neurog2* locus was induced, while there was no induction in the DMSO-treated control sister retinas (Figure S2). The shift from Müller glial fate (Sox9+/Ccnd3+) to progenitor fate (Sox9+/Ascl1+/Neurog2) was not accompanied by a loss of the cells, since the number of Sox9+ cells remained the same, but rather the inhibition of Notch signaling caused the Müller glia to express markers of retinal progenitors, even at postnatal day 12, nearly a week after the cells have exited the cell cycle.

**Figure 9:**
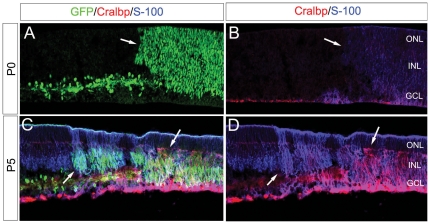
Notch signaling promotes glial character. A–D, Staining for GFP (green) indicates regions of ROSA-NICD activation (arrows). Noted that GFP is also expressed in a subset of amacrine cells, driven under the alphaPax6 promoter. A–B, Staining for the glial markers Cralbp (red) and S-100 (blue) shows a small upregulation of these markers in NICD-expressing progenitor cells at P0. C–D, Staining for the glial markers Cralbp (red) and S-100 (blue) shows a dramatic upregulation of these markers in NICD-expressing glial cells at P5, when compared to adjacent wild type Müller glia.

We also used a genetic approach to determine the effects of activating the Notch pathway on Müller glial differentiation at the stages of retinal development when the other gliogenic signals are increasing. For these experiments, we generated *αPax6cre*; *ROSA-NICD* mice, in which cre-mediated recombination leads to constitutively activated Notch from the intracellular domain of the Notch1 gene [32] Previous studies using a *Chx10-cre/ROSA-NICD* line of mice demonstrated that NICD overexpression promotes Müller glial gene expression [10] We therefore analyzed the mice at P0, when the gliogenic signals are not yet highly expressed, and at later postnatal ages, when these signals are increasing. Areas with active Notch signaling can be visualized with the IRES nuclear GFP reporter that is co-transcribed with the NICD (Figure 9A, arrowhead), and at both ages there is an increase in the expression of glial markers; however, there is a clear difference in the level of expression of glial markers in the NICD-expressing regions between the P0 and P5 retinas. At P0, NICD-expressing progenitors express only low levels of the glial markers Cralbp and S-100 and are still mitotic (data not shown), suggesting they have not yet begun differentiation as post-mitotic glial cells. By contrast, at P5, the regions of the retinas that express the NICD transgene show very intense labeling for the Müller glial markers Cralbp and S-100, with levels exceeding those of adjacent wild type Müller glia (Figure 9A and B). These data support a developmental switch in the role of Notch signaling in gliogenesis in the retina (Figure 9C and D).

## Discussion

In this study, we have carried out the first genome-wide analysis of glial development. We were able to take advantage of a Hes5-GFP line of mice to selectively purify retinal progenitors and Müller glia continuously through the developmental transition. The results of the analysis show the rapid decline in genes associated with the mitotic cell cycle in the first postnatal week, a more gradual decline in the progenitor genes associated with their neurogenic potential (*Ascl1*, *Neurog2*) and the gradual increase in several genes relevant to glial differentiation (*Egfr*, *Cntfr*, *Bmpr1b*). Surprisingly, however, the components of the Notch pathway did not decline like the proneural genes, but rather remained highly expressed, and even reached their peak, in the second postnatal week. This was true for *Hey2*, *Hes1*, *Hes5* and all of the *Id* family. We therefore tested whether Notch continues to be required in the postmitotic glial cells. We found that inhibition of Notch signaling up to a week after the cells have exited the mitotic cycle leads to a loss in Müller cell markers, and a reacquisition of the progenitor phenotype. By contrast, activating Notch signaling in progenitors leads to their expression of glial markers; while this effect is minimal at P0, at later postnatal ages when gliogenic factors are at higher levels in the retina, but expression of the Notch-ICD causes a dramatic increase in Müller glial-specific gene expression. Our results thus demonstrate a continuing requirement for Notch signaling in the stabilization and maintenance of the Müller glial fate for almost a week after the cells have exited the mitotic cell cycle, during the period of postnatal development when the other gliogenic signals are increasing. After this sensitive period, the Müller cells are no longer dependent on Notch signaling to maintain their identity, suggesting that other mechanisms now take over to repress the proneural gene expression.

Comparison of the Müller glial-enriched genes in our microarray analysis with those of CNS glia, revealed that Müller glia share many genes in common with astrocytes. Over 40% of the most highly expressed astrocyte-specific genes in one analysis [33] were present in the Müller-enriched clusters (1 and 10), while less that 2% of the oligodendrocyte enriched genes were present in our Müller-enriched gene set. Although a previous single-cell microarray analysis of the Müller glial transcriptome failed to reveal a common gene cluster with either astrocytes or oligodendrocytes [19], these investigators suggested this might be due to technical limitations with the single-cell technique. As shown in Table 1, although some of the more common astrocyte-specific genes are not expressed in the FACS purified Müller glia (eg. GFAP), we found that over 20 genes expressed in astrocytes are also highly enriched in Müller glia, and thus we conclude that Müller glia and astrocytes are indeed related glial cell types. The conclusion that Müller glia resemble astrocytes is further strengthened by developmental data suggesting that the same molecular mechanisms that promote the astrocyte fate from CNS progenitors also promote Müller glial development from retinal progenitors. The astrocyte promoting factors CNTF and EGF are also potent Müller glial-promoting factors [12], [15]. Notch signaling also promotes both astrocytes and Müller glia (for review see [7], [8]). Transcription factors that direct progenitors to the glial fate are also conserved between Müller glia and astrocytes: Hes5 and Hes1 are important for the development of both astrocytes and Müller glia [6], [34], [35]. Additional transcription factors that are involved in glial development were also revealed in our analysis. Sox2, Sox9, Nfi-a/b/c/x and Id family genes were all upregulated in the Müller glia from day 7 to day 21 as they matured. In fact, the majority of transcription factors associated with astrocytes in the Cahoy et al [33] study are also upregulated during Müller glial development. One particular family of genes, the Klf family, is particularly well represented in Müller glia, with Klf 4,6, 9,10, and 15 all upregulated during Müller glial maturation, while only Klf15 is increased in astrocytes.

**Figure 10:**
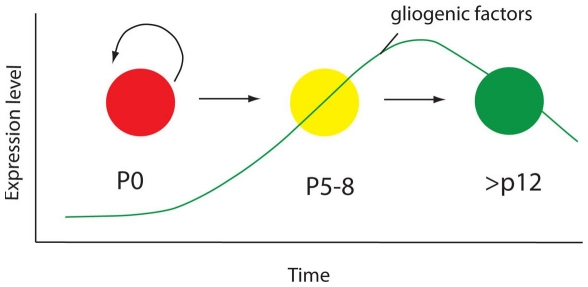
Model for gliogenesis in the retina. As the last progenitors exit the cell cycle (red), a subset of the postmitotic progeny are committed to the glial fate. These presumptive Müller glia (yellow) retain Notch signaling and are prevented from differentiating into bipolar neurons or rod photoreceptors; however, their glial identity is labile, and it is only with the increase in the gliogenic signals over the next week that secondary mechanisms stabilize the glial fate (green).

As noted above, it is well established that Notch signaling promotes the glial fate from progenitors, at least in part from the Hes1/5 mediated inhibition of the proneural genes [7]. In addition, the prolonged expression of Notch signaling components in glia has been previously noted in a similar microarray study of CNS neuronal and glial development [33]. They suggested that Notch might have a role in maintaining astrocyte cell fate. We have tested this possibility directly for Müller glia and find that at least for the first week after these cells become postmitotic, as they are maturing, Notch signaling is indeed required to maintain their fate. Thus, it is possible that a similar requirement exists for astrocytes. However, in Müller glia, this requirement is lost after two weeks of postnatal development, suggesting that other mechanisms likely exist to maintain Müller fate in mature mice. Concomitant with the sensitive period for Notch signaling in the postmitotic Müller glia, there is a steady increase in the receptors for the other “gliogenic” signals, including *Cntfr*, *Egfr* and *Bmpr1b*. These signals are important in promoting the Müller glial fate in retina and the astrocyte fate in the rest of the CNS (Figure 10). Our data, taken together, suggest the following model for gliogenesis in the retina. As the last progenitors exit the cell cycle at P4 (in central retina), the gliogenic signals have begun to rise and so a subset of the postmitotic progeny are committed to the glial fate. These presumptive Müller glia retain Notch signaling and are prevented from differentiating into bipolar neurons or rod photoreceptors; however, their glial identity is labile, and it is only with the increase in the gliogenic signals over the next week that secondary mechanisms (perhaps involving Notch dependent DNA methylation of progenitor genes) stabilize the glial fate.

The model leaves several questions. Why do non-glial postmitotic progeny, eg. rod photoreceptors, downregulate Notch signaling components, whereas the developing Müller glia retain them? What drives the expression of the receptors for the gliogenic factors as the progenitors transition to Müller glia and as the Müller glia mature? What are the mechanisms that stabilize the Müller glial fate so that Notch is no longer required after postnatal day 12? A better understanding of these mechanisms might enable reprogramming of these cells to retinal progenitors as a basis for regeneration after damage, as occurs in non-mammalian vertebrates.

## Materials and Methods

### Animals

Mice were housed in the University of Washington Department of Comparative Medicine and the University of Washington Institutional Animal Care and Use Committee (UW-IACUC) approved the animal housing and the experimental protocols used in this study. (Protocol # 2448-08/approval 2/22/11). Hes5-GFP transgenic mice were generated using a 3 kb portion of the *Hes5* gene, including 1.6 kb of the 5-prime flanking region, with eGFP cloned into the translational start site [21]. Alpha-Pax6-Cre, *Neurog2^ki-CreER^*, *Rosa26LacZ*, *Rosa26YFP*, *Rosa26^NICD^* mice have been described [32], [36], [37], [38], [39]. *ROSA-NICD* mice were obtained from Jackson Laboratories (Bar Harbor, ME), and genotyped according to supplier's protocols. These were crossed to *αPax6cre* mice obtained from Ruth Ashery-Padan (Tel-Aviv University, Tel-Aviv, Israel) to generate *αPax6cre*; ROSA-NICD mice. Embryos were collected from timed pregnant homozygous wild type C57/BL6 or Hes5-GFP mice Postnatal day 0 (P0) was defined as the day of birth.

### FACS purification and Affymetrix Analysis

To isolate the cells that express Hes5-GFP, we dissociated them into a single-cell suspension using papain. Prior to sorting, aggregates were removed by passing through a 40 um cell strainer. FACS was carried out on BD Aria II sorter, gated for a high level of GFP expression. We used a minimum of 5 mice per time point, and collected a minimum of 10∧6 GFP+ cells. Immediately following sorting, the GFP+ cells were lysed in Trizol (Invitrogen) and stored at −80°C. Total RNA was extracted from the cells by phenol-chloroform extraction and ethanol precipitation as per manufacturer's instructions. The mRNA was extracted from the cells recovered from the FACS and cDNA was generated for Affymetrix analysis without amplification. This was followed by DNAse-1 (Qiagen) treatment followed by RNA cleanup using Qiagen RNA mini cleanup kit. The RNA was then checked for integrity and run as per manufacturer's guidelines on the Mouse Gene 1.0 ST microarray (Affymetrix) at the Institute for Systems Biology (Seattle). The RIN numbers for the samples ranged from 8–10 with a mean of 8.98. The microarray data was normalized with Affymetrix Power Tools software and subjected to K-medians clustering using the Cosine Correlation Distance Metric to subdivide the gene set into 10 different clusters based on the expression profiles between the different groups using TM4 Multi-Experiment Viewer software [40], [41]. In some cases, RNA from the FACS purified progenitors and glia was also used to make cDNA using Superscript II RT kit (Invitrogen) as previously described and quantitative real time PCR was performed following *glyceraldehyde-3-phosphate dehydrogenase* GAPDH normalization [30]. All primer sequences for QPCR were obtained from PrimerBank. The primers used for PCR are listed in Table S3.

### In Situ Hybridization and Immunolabeling

In situ hybridization was carried out as described in previous publications using digoxigenin-labeled probes for Hes5, Notch1, Dll1, Dll3 and Neurog2 [30]. Eyes were fixed overnight at 4°C in modified Carnoy's solution (60% ethanol, 11.1% formaldehyde (30% of 37% stock), 10% glacial acetic acid), embedded in paraffin and sectioned at 6–8 µm. Slides were hybridized with probe overnight hybridization and hybridized probe was detected using anti-Digoxygenin alkaline phosphatase conjugated antibody (1∶2000 dilution, Roche Biochemical, Indianapolis, IN). After in situ hybridization, sections were post-fixed in 4% PFA and rinsed in PBS prior to immunofluorescent labeling.

For the immunofluorescent labeing, we used the following primary antibodies: chicken anti-GFP (1∶500 dilution, Abcam, USA, Cat. No. AB13970); goat anti-Sox2 (1∶500, Santa Cruz Biotechnology, USA, Sox2 Y-17 Cat. No. SC-17320); Cralbp (rabbit, UW55, kindly provided by J. Saari); Sox9 (rabbit, Chemicon); Id1 (sc488, Santa Cruz; TuJ1 (mouse, Covance); Glast (guinea pig, Chemicon); BrdU (rat, Accurate); mouse anti-S-100 (1∶500, Sigma-Aldrich, St. Louis, MO). Primary antibodies were diluted in block and incubated overnight at 4°C. Slides were then washed in PBS 3×10 min, and incubated in species-specific fluorescent-labeled secondary antibodies AlexaFluor 488, 568, or 594 nm (1∶500, Invitrogen). After immunostaining, slides were coverslipped in Fluoromount G (Southern Biotechnology, Birmingham, AL). Whole-mount immunolabeling was performed as described [30]. Labeled sections were imaged on a Zeiss Axioplan 2 microscope or a Zeiss LSM Pascal confocal microscope. Multiphoton microscopy was used to obtain high resolution images of postnatal and adult *Hes5GFP* retinas that were dissected, fixed briefly, sliced, mounted in agarose, and scanned at 890 nm (25× Ultra-Objective, 1.05NA, FV1000 MPM Olympus).

Immunolabeling for activated Notch1 and Hes1 was performed as described [30]. Briefly, eyes were fixed overnight at 4°C in modified Carnoy's solution, dehydrated though an EtOH series, prepared for paraffin embedding, and sectioned at 6–8 µm. Slides were baked overnight at 68°C, dewaxed in Xylene, rinsed in 100% EtOH, and air-dried at room temperature. Antigen retrieval was accomplished by autoclave treatment (5 min, 105°C) in TE buffer (10 mM TrisCl, 1 mM EDTA, pH 9.0). Sections were washed with PBS, blocked in 10% goat serum in PBT for 1 h, incubated with rabbit anti-actNotch1antibody overnight, washed 4× with PBS, incubated with goat-anti rabbit alkaline phosphatase (1∶500, Sigma) for 1 h, washed 4× with PBT, equilibrated with NTMT, pH 9.0, and incubated in NBT/BCIP substrate (Sigma). Sections were washed in PBS and subjected to sequential immunolabeling and fluorescent detection with primary and secondary antibodies as described below, followed by DAPI counterstaining and mounting.

### Acute pharmacological inhibition of canonical Notch signaling

The γ-secretase inhibitor N-[N-(3,5-difluorophenacetyl)-l-alanyl]-S-phenylglycine t-butyl ester (DAPT; Sigma) was used to inhibit gamma-secretase dependent Notch signaling as described [30]. Briefly, sister wildtype retinas were dissected at the given age, cultured separately in the presence of 10 µM DAPT or DMSO vehicle control for 2DIV, fixed briefly, sectioned, and immunolabeled. Changes in Ascl1/Ccnd3 expression in Sox9+ Müller glia were quantified as numbers of double positive cells per field, Student's T-test was used to evaluate significance, P<0.05 considered significant. For genetic induction of *Neurog2^ki-CreER+/−:YFP^* in postmitotic Müller glia experiments, retina culture media was also supplemented to 1 µM tamoxifen (Sigma) to activate the inducible Cre for recombination of LacZ or YFP floxed reporters (n = 5 pairs per age, 5/5 DAPT-treated retinas induced *Neurog2* activity from this locus).

## Supporting Information

Figure S1 **Unsupervised K-medians clustering of all genes on the array (28,853) was used to generate 10 clusters, and the gene expression levels (log transformed) across the five ages of retinal development are shown for each cluster.** The overall trend of the genes in each cluster, as well as the relative level of expression is shown. The number of genes in each cluster is also given for each graph.
(TIF)

Figure S2 **Genetic induction of** ***Neurog2*** **in retinal progenitors and postmitotic Muller glia.** (**A**) Experimental strategy to detect *Neurog2* activity based on tamoxifen-inducible CreER knock-in mice (*Neurog2^ki-CreER^*, Zirlinger et a., 2002) were crossed with Rosa26-stop-LacZ (not shown) and Rosa26-stop-YFP floxed reporter mice (Soriano, 1999; Srinivas et al.,2001): note that due to the knock-in, *Neurog2* wildtype mice (*Neurog2^ki-CreER−/−^*) will not report, and that homozygous CreER knock-in mice are functional *Neurog2* knockouts (*Neurog2^ki-CreER+/+^*). Postnatal day (P0, B–C) and P12 (E, F) sister retinas were collected and cultured for 2 days *in vitro* (DIV), both in the presence of tamoxefin (TM), one retina was treated with DAPT, whereas the sister retina was treated with DMSO control, fixed, and stained as wholemounts with anit-Sox9 and anti-GFP antibodies (for Rosa YFP reporter, Rosa LacZ not shown). Laser scanning confocal microscopy (LSCM) was used to image central retinal explants *en face*. At P0, acute TM-mediated labeling reveals *Neurog2* activity in a subset of Sox9+ progenitors, which increases upon acute Notch inhibition (compare sister retinas, C). In P12 retinas, *Neurog2* has been downregulated in postmitotic Sox9+ Muller glia, but acute DAPT-treatment can still induce *Neurog2* activity (E, F; similar results at P8, not shown). Scale bar = 75 µn B–C, 50 µn E–F.
(JPG)

Table S1 The number of genes that fell into each of the 10 clusters after unsupervised K-medians clustering of all genes on the array across the five ages of retinal development.
(DOCX)

Table S2 Genes present in Cluster 2, which are expressed in retinal progenitors (P0) but not in Hes5-GFP+ Muller glia at P7 or after, associated with the mitotic cell cycle by GO analysis. These are likely to be retinal progenitor enriched genes.
(DOCX)

Table S3 The primers used for PCR. All primer sequences for QPCR were obtained from PrimerBank.
(DOCX)

Table S4 The results of the unsupervised K-medians cluster analysis for all genes, sorted into the 10 clusters.
(XLSX)
