# Supplementary figures and images for: Genome-Wide Analysis of Müller Glial Differentiation Reveals a Requirement for Notch Signaling in Postmitotic Cells to Maintain the Glial Fate
Source: PLoS One. 2011 Aug 2;6(8):e22817. doi: 10.1371/journal.pone.0022817 (PMC3149061; doi:10.1371/journal.pone.0022817)

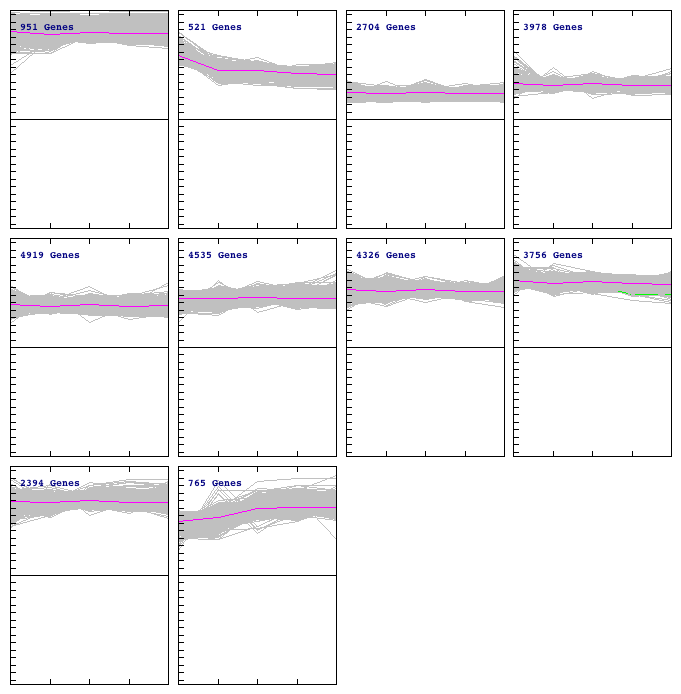

Supplement: Figure S1 — Unsupervised K-medians clustering of all genes on the array (28,853) was used to generate 10 clusters, and the gene expression levels (log transformed) across the five ages of retinal development are shown for each cluster. The overall trend of the genes in each cluster, as well as the relative level of expression is shown. The number of genes in each cluster is also given for each graph. (TIF) [file pone.0022817.s001.tif]

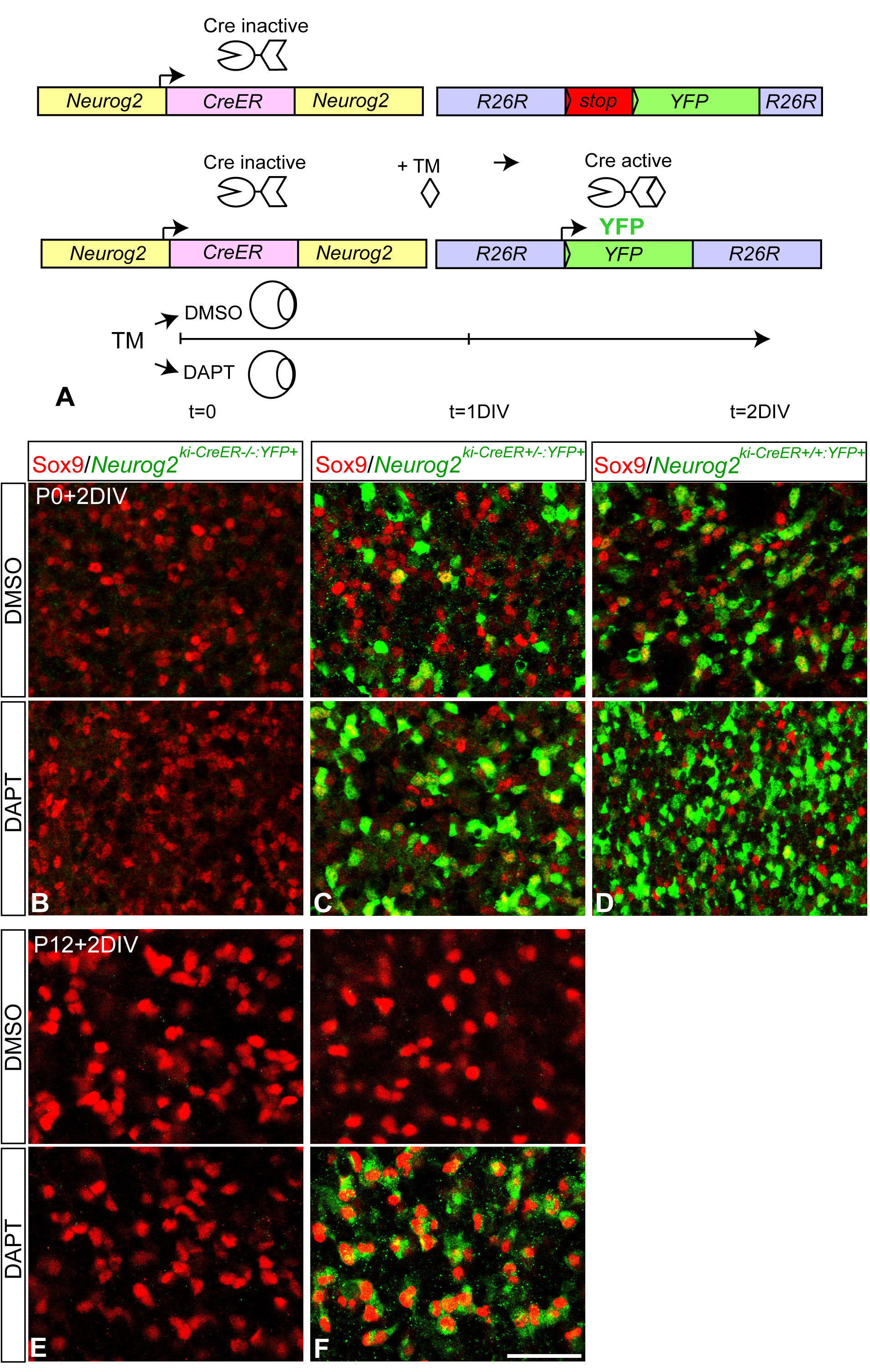

Supplement: Figure S2 — Genetic induction of Neurog2 in retinal progenitors and postmitotic Muller glia. (A) Experimental strategy to detect Neurog2 activity based on tamoxifen-inducible CreER knock-in mice (Neurog2ki-CreER, Zirlinger et a., 2002) were crossed with Rosa26-stop-LacZ (not shown) and Rosa26-stop-YFP floxed reporter mice (Soriano, 1999; Srinivas et al.,2001): note that due to the knock-in, Neurog2 wildtype mice (Neurog2ki-CreER−/−) will not report, and that homozygous CreER knock-in mice are functional Neurog2 knockouts (Neurog2ki-CreER+/+). Postnatal day (P0, B–C) and P12 (E, F) sister retinas were collected and cultured for 2 days in vitro (DIV), both in the presence of tamoxefin (TM), one retina was treated with DAPT, whereas the sister retina was treated with DMSO control, fixed, and stained as wholemounts with anit-Sox9 and anti-GFP antibodies (for Rosa YFP reporter, Rosa LacZ not shown). Laser scanning confocal microscopy (LSCM) was used to image central retinal explants en face. At P0, acute TM-mediated labeling reveals Neurog2 activity in a subset of Sox9+ progenitors, which increases upon acute Notch inhibition (compare sister retinas, C). In P12 retinas, Neurog2 has been downregulated in postmitotic Sox9+ Muller glia, but acute DAPT-treatment can still induce Neurog2 activity (E, F; similar results at P8, not shown). Scale bar = 75 µn B–C, 50 µn E–F. (JPG) [file pone.0022817.s002.jpg]
