# Supplementary material for: Genome-Wide Analysis of Müller Glial Differentiation Reveals a Requirement for Notch Signaling in Postmitotic Cells to Maintain the Glial Fate
Source: PLoS One. 2011 Aug 2;6(8):e22817. doi: 10.1371/journal.pone.0022817 (PMC3149061; doi:10.1371/journal.pone.0022817)
Supplement: Table S1 — The number of genes that fell into each of the 10 clusters after unsupervised K-medians clustering of all genes on the array across the five ages of retinal development. (DOCX) [file pone.0022817.s003.docx]

Table 1.

| Cluster # | Percent of total | Genes in cluster |
| --- | --- | --- |
| 1 | 3 | 951 |
| 2 | 2 | 521 |
| 3 | 9 | 2704 |
| 4 | 14 | 3978 |
| 5 | 17 | 4919 |
| 6 | 16 | 4535 |
| 7 | 15 | 4326 |
| 8 | 13 | 3756 |
| 9 | 8 | 2394 |
| 10 | 3 | 765 |

K-medians unsupervised cluster analysis of Affymetrix expression levels for all ages. Genes were assigned to cluster based on their pattern of expression across the period of analysis.
