# Supplementary material for: Genome-Wide Analysis of Müller Glial Differentiation Reveals a Requirement for Notch Signaling in Postmitotic Cells to Maintain the Glial Fate
Source: PLoS One. 2011 Aug 2;6(8):e22817. doi: 10.1371/journal.pone.0022817 (PMC3149061; doi:10.1371/journal.pone.0022817)
Supplement: Table S2 — Genes present in Cluster 2, which are expressed in retinal progenitors (P0) but not in Hes5-GFP+ Muller glia at P7 or after, associated with the mitotic cell cycle by GO analysis. These are likely to be retinal progenitor enriched genes. (DOCX) [file pone.0022817.s004.docx]

Table 4

| 2610016C23Rik | Chek1 | Kif2c | Rrm2 |
| --- | --- | --- | --- |
| 4632434I11Rik | Chek2 | Kif4 | Rtkn2 |
| 4922501C03Rik | Cit | Kntc1 | Sass6 |
| Anln | Ckap2 | Lin9 | Sgol1 |
| Asf1b | Cks2 | Mad2l1bp | Sgol2 |
| Aspm | Clspn | Mapk12 | Smc2 |
| Aurka | D2Ertd750e | Mbd4 | Spag5 |
| Aurkb | Dbf4 | Mcm10 | Spc24 |
| Birc5 | Ddx11 | Mcm3 | Ssbp1 |
| Blm | Dlg7 | Mcm5 | Syce2 |
| Brca1 | Dna2l | Mcm6 | Tcp1 |
| Brca2 | Dock11 | Mcm8 | Tk1 |
| Bub1 | Dsn1 | Mdc1 | Top2a |
| Bub1b | Dtl | Mns1 | Tpx2 |
| Ccna2 | E2f1 | Mphosph1 | Ube2c |
| Ccnb1 | E2f2 | Mtap | Uhrf1 |
| Ccnb2 | E2f7 | Ncapg2 | Ung |
| Ccne1 | E2f8 | Ncaph | Xrcc2 |
| Ccnf | Ect2 | Ndc80 | Zwilch |
| Cdc20 | Esco2 | Neil3 |  |
| Cdc25a | Espl1 | Nek2 |  |
| Cdc25b | Fanca | Nsl1 |  |
| Cdc25c | Fancb | Nuf2 |  |
| Cdc2a | Fancd2 | Nusap1 |  |
| Cdc45l | Fancl | Pbk |  |
| Cdc6 | Fancm | Plk1 |  |
| Cdc7 | Fbxo5 | Pmf1 |  |
| Cdca2 | Fignl1 | Pola1 |  |
| Cdca3 | Fmn2 | Pola2 |  |
| Cdca5 | Foxm1 | Pole |  |
| Cdca7 | Gen1 | Polh |  |
| Cdca8 | Gins1 | Polq |  |
| Cdt1 | Gtse1 | Prc1 |  |
| Cenpa | Incenp | Prim1 |  |
| Cenpe | Kif14 | Prim2 |  |
| Cenpf | Kif15 | Racgap1 |  |
| Cenpn | Kif20a | Rad18 |  |
| Cep55 | Kif22 | Rad51 |  |
| Chaf1a | Kif24 | Rad51l1 |  |
| Chaf1b |  | Rbbp8 |  |

Genes associated with the mitotic cell cycle by GO analysis, which are expressed in retinal progenitors but not in Hes5-GFP+ Muller glia at P7 or after. These are likely to be retinal progenitor enriched genes.
