# Supplementary material for: Genome-Wide Analysis of Müller Glial Differentiation Reveals a Requirement for Notch Signaling in Postmitotic Cells to Maintain the Glial Fate
Source: PLoS One. 2011 Aug 2;6(8):e22817. doi: 10.1371/journal.pone.0022817 (PMC3149061; doi:10.1371/journal.pone.0022817)
Supplement: Table S3 — The primers used for PCR. All primer sequences for QPCR were obtained from PrimerBank. (DOCX) [file pone.0022817.s005.docx]

**Dll3**

**PrimerBank ID 6681199a1**

**Amplicon Size 199**

**Sequence (5' -> 3') Length Tm Location**

**Forward Primer CTGGTGTCTTCGAGCTACAAAT 22 60.0 74-95**

**Reverse Primer TGCTCCGTATAGACCGGGAC 20 62.7 272-253**

**Dll1**

**PrimerBank ID 6681197a1**

**Amplicon Size 168**

**Sequence (5' -> 3') Length Tm Location**

**Forward Primer CAGGACCTTCTTTCGCGTATG 21 60.8 165-185**

**Reverse Primer AAGGGGAATCGGATGGGGTT 20 62.9 332-313**

**FoxN4**

**PrimerBank ID 21654746a1**

**Amplicon Size 272**

**Sequence (5' -> 3') Length Tm Location**

**Forward Primer CATGAAGGAGCACTTCCCCTA 21 60.9 678-698**

**Reverse Primer TTTCCGGGCGGTCTGAGAT 19 63 949-931**

**Olig2**

**PrimerBank ID 8393874a1**

**Amplicon Size 90**

**Sequence (5' -> 3') Length Tm Location**

**Forward Primer TCCCCAGAACCCGATGATCTT 21 62.4 40-60**

**Reverse Primer CGTGGACGAGGACACAGTC 19 62 129-111**

**Glast (Slc1a3)**

**PrimerBank ID 24233554a1**

**Amplicon Size 144**

**Sequence (5' -> 3') Length Tm Location**

**Forward Primer ACCAAAAGCAACGGAGAAGAG 21 60.5 4-24**

**Reverse Primer**

**GGCATTCCGAAACAGGTAACTC, 22 61.2 147-126**

**CRALBP (Rlbp1)**

**PrimerBank ID 10181110a1**

**Amplicon Size 162**

**Sequence (5' -> 3') Length Tm Location**

**Forward Primer GGCACTTTCCGCATGGTTC 19 61.7 16-34**

**Reverse Primer CCGGGTCTCCTCCTTTTCAT 20 61.0 177-158**
